# Oral health indices and microbiota populations of adult cats consuming wet or dry diets

**DOI:** 10.3389/fvets.2025.1678016

**Published:** 2025-11-25

**Authors:** Patrícia M. Oba, Olivia R. Swanson, Gene Pavlovsky, Chloe R. Dupleix, Stephanie C. J. Sharping, Kelly S. Swanson

**Affiliations:** 1 Department of Animal Sciences, University of Illinois Urbana-Champaign, Urbana, IL, United States; 2 Department of Veterinary Clinical Medicine, College of Veterinary Medicine, University of Illinois Urbana-Champaign, Urbana, IL, United States; 3 Division of Nutritional Sciences, University of Illinois Urbana-Champaign, Urbana, IL, United States

**Keywords:** plaque microbiota, oral health, microbiota modulation, biofilm, microbial diversity

## Abstract

Oral microbiota play a critical role in feline periodontal disease, with wet diets being associated with poor oral health. Because the oral microbial communities of cats remain underexplored, this study aimed to evaluate differences in the oral health indices and microbiota of cats fed a dry or wet diet. Twenty healthy adult cats had their teeth cleaned and polished. Cats were randomly allotted to a dry or wet diet and fed for 28 weeks. At that time, sulfur-containing compound concentrations and salivary pH were measured, plaque, calculus and gingivitis scores were assessed by a blinded veterinarian, and supragingival and subgingival plaque samples were collected for microbiota analysis. Microbiota data were evaluated using QIIME2. All other data were analyzed using the Mixed Models procedure of SAS. Cats fed the dry diet had lower tooth calculus coverage and thickness than cats fed the wet diet. Gingivitis scores were higher in cats fed the wet diet than those fed the dry diet. Other clinical measures did not differ. Bacterial alpha diversity measures on supragingival plaque were lower in cats fed the wet diet than those fed the dry diet. Bacterial beta diversity measures revealed distinct microbial communities between diet groups, with numerous changes to bacterial phyla and genera relative abundances. Compared with cats fed the dry diet, cats fed the wet diet had higher relative abundances of Bacteroidota and *Bacteroides* in supragingival samples and greater relative abundances of Synergistota, *Bacteroides*, *Fretibacterium*, *Campylobacter*, and *Christensenellaceae R-7* group in subgingival samples. In contrast, cats fed the dry diet had higher relative abundances of Proteobacteria, *Streptococcus*, *Luteimonas*, *Lautropia* in supragingival plaque than those cats fed the wet diet. Although most clinical indices did not differ between groups, the reduced calculus scores, enrichment of health-associated bacteria and reduction in disease-associated bacteria suggest oral health benefits of dry diets.

## Introduction

Dietary composition and physical characteristics can influence the development and progression of periodontal disease in companion animals. Among these factors, the texture of the diet, particularly its abrasive properties, is a key mechanism by which diet can influence oral health by reducing dental plaque accumulation (1). Supporting this, a recent study in dogs showed that plaque coverage tended to be higher in animals fed wet versus dry food, although other oral health scores did not differ (2). Dietary characteristics, including texture and nutrient composition, can directly affect the oral environment by maintaining oral tissue integrity, influencing the metabolic activity of plaque bacteria, promoting salivary secretion, and providing mechanical cleaning of teeth and oral surfaces through physical contact (3). As a result, dry, crunchy commercial diets are widely recognized for their potential oral health benefits in both dogs and cats. This concept is supported by early studies showing that dogs fed hard or solid diet maintained healthier teeth and gingiva than those consuming the same diet in a ground or minced form (4–6). Notably, even limited chewing, such as that involved in eating minced diet, provided some cleansing effect compared with no mastication at all, as demonstrated in dogs fed via gastric intubation (7).

Despite the widespread belief that dry diets promote oral health, there is limited scientific evidence supporting this claim, particularly in cats. Some studies suggest potential benefits; for instance, dental calculus and plaque were reported to be less frequent in cats fed dry diets than those fed wet diets (8). A cross-sectional study of 41 client-owned cats further reported that the likelihood of poor oral health was lower in young and adult cats consuming dry diets than in older cats fed wet diets, with diet type exerting a stronger effect on oral health than age itself (9). Complementing these findings, a small pilot study (*n* = 10) demonstrated that cats exclusively fed dry diets exhibited greater oral bacterial diversity and higher relative abundances of *Porphyromonas* and *Treponema* than those fed wet diets (10). Despite these observations, controlled studies directly evaluating the impact of diet type on the feline oral microbiota and associated health outcomes are still lacking, highlighting the need for further research in this area.

This research evaluated the impact of diet type (dry vs. wet) on oral health outcomes and plaque composition in healthy adult cats over a 28-week period, using a clean mouth model. This approach, initiating the study after professional dental cleaning, allowed for a standardized oral health baseline, minimizing confounding effects from pre-existing dental conditions. To reduce environmental variability, the trial was conducted under controlled laboratory conditions. We hypothesized that cats consuming a dry diet would have better oral health outcomes and a more beneficial oral microbiota composition than those fed a wet diet.

## Materials and methods

All animal care procedures were approved by the University of Illinois Institutional Animal Care and Use Committee prior to initiation of the experiment (protocol #24025).

### Animals, housing, and diets

Twenty adult American Shorthair cats (12 females and 8 males; 6.28 ± 0.29 yr.; 4.18 ± 0.15 kg body weight; 5.25 ± 0.05 body condition score) were used in a longitudinal completely randomized design study. Cats were housed individually in cages (1.02 m deep, 0.76 m wide; 0.71 m high) during feeding times (8–10 a.m.) in a temperature- and light-controlled (14-h light, 10-h dark cycle) room in the Edward R. Madigan Animal Facility at the University of Illinois Urbana-Champaign. At other times, cats were group-housed and able to socialize and exercise outside of their cages. Cages and the group-housing area were cleaned daily. Cats were allowed access to various toys and scratching poles for environmental enrichment and play time with human interaction at least 2 times per week. Water was available ad libitum. Body weight was measured at the beginning and end of study, and were assessed weekly during the study. Body condition scores were assessed using a 9-point scale (11) weekly prior to the morning feeding.

Cats were assigned to 1 of 2 diets to balance sexes between treatments: dry diet (Purina - Cat Chow Complete with Real Chicken Dry Cat Food, *n* = 10; 4 males and 6 females) or wet diet (Purina - Friskies Pate Country Style Dinner Canned Cat Food, *n* = 10; 4 males and 6 females; Table 1). Both dietary treatments tested were commercial diets formulated to meet all Association of American Feed Control Officials (12) nutrient recommendations for adult cats at maintenance. Daily food intake was recorded. Individual food intake was determined by placing the animals in separate cages during feeding time. The food was weighed before and after each meal to accurately measure consumption. All cats were fed to maintain body weight throughout the study.

**Table 1:** Guaranteed analysis and ingredient list of diets.

Item	Dry^1^	Wet^2^
Caloric content, kcal/g	3.80	1.16
Moisture (max.), %	12.0	78.0
	Dry matter basis	
Crude protein (min.), %	36.36	45.45
Crude fat (min.), %	13.64	22.73
Crude fiber (max.), %	3.41	4.55

^1^Purina - Cat Chow Complete with Real Chicken Dry Cat Food (Manufactured by Nestle Purina PetCare Company, St. Louis, MO). Ingredients: Chicken By-Product Meal, Ground Yellow Corn, Corn Gluten Meal, Whole Grain Wheat, Rice, Soy Flour, Beef Fat Preserved With Mixed-Tocopherols, Chicken, Fish Meal, Liver Flavor, Phosphoric Acid, Calcium Carbonate, Salt, Potassium Chloride, Choline Chloride, Minerals [Zinc Sulfate, Ferrous Sulfate, Manganese Sulfate, Copper Sulfate, Calcium Iodate, Sodium Selenite], Taurine, Vitamins [Vitamin E Supplement, Niacin (Vitamin B-3), Vitamin A Supplement, Calcium Pantothenate (Vitamin B-5), Thiamine Mononitrate (Vitamin B-1), Riboflavin Supplement (Vitamin B-2), Vitamin B-12 Supplement, Pyridoxine Hydrochloride (Vitamin B-6), Folic Acid (Vitamin B-9), Vitamin D-3 Supplement, Biotin (Vitamin B-7), Menadione Sodium Bisulfite Complex (Vitamin K)], Dl-Methionine, Red 40, Yellow 5, Blue 2.

^2^Purina - Friskies Pate Country Style Dinner Canned Cat Food (Manufactured by Nestle Purina PetCare Company, St. Louis, MO). Ingredients: Meat By-Products, Water, Chicken, Poultry By-products, Rice, Artificial And Natural Flavors, Guar Gum, Added Color, Potassium Chloride, Salt, Carrageenan, Choline Chloride, Vitamins [Thiamine Mononitrate (Vitamin B-1), Vitamin E Supplement, Niacin (Vitamin B-3), D-calcium Pantothenate (Vitamin B-5), Pyridoxine Hydrochloride (Vitamin B-6), Riboflavin Supplement (Vitamin B-2), Vitamin A Supplement, Biotin (Vitamin B-7), Vitamin d-3 Supplement, Vitamin B-12 Supplement, Folic Acid (Vitamin B-9)], Taurine, Minerals [Ferrous Sulfate, Zinc Oxide, Copper Proteinate, Sodium Selenite, Manganese Sulfate, Potassium Iodide], Sodium Tripolyphosphate.

### Experimental timeline

Before cats were allotted to treatments, a board-certified veterinarian polished and cleaned the teeth. After dental cleaning, cats were randomly allotted to a commercial dry extruded kibble diet or commercial wet canned diet and fed for 28 weeks. After 28 weeks on study, breath samples, salivary pH, and plaque samples were collected and analyzed. On week 28, gingivitis, plaque, and calculus scoring were conducted by a board-certified veterinarian that was blinded to treatments.

### Oral sample collection and scores

Cats were transported to the Veterinary Teaching Hospital on the University of Illinois campus for oral sample collection and scoring. All cats had food withheld overnight, with free access to water until the morning of the procedure. First, the hair over the right cephalic vein was clipped, topical 5% lidocaine cream was applied to the catheter site, and cats were premedicated with an intramuscular injection of butorphanol (0.4 mg/kg) and alfaxalone (2 mg/kg). After 15 min, the catheter site was aseptically prepared, and a 22-gauge intravenous catheter was placed in the cephalic vein. Following catheterization, cats were administered maropitant (1 mg/kg) or physiologic saline IV and anesthesia was induced with propofol administered to effect. Topical lidocaine (0.2 mL, 2% lidocaine) was then applied to the vocal folds, cats were orotracheally intubated, and anesthesia was maintained with isoflurane (1–2%) delivered in oxygen. Intravenous fluids (Normosol-R) were delivered at 3 mL/kg/h throughout the maintenance phase of anesthesia, and vital parameters were monitored continuously using a multiparameter monitor. All anesthetic events were supervised by a board-certified veterinary anesthesiologist.

### Oral sulfur-containing compounds

Halimeter measurements were conducted approximately 12 h after feeding and obtained for each cat using a clean plastic straw (i.e., a clean straw was used for each measurement) as an extension of the halimeter air drawing hose. The highest reading of volatile sulfur compounds over a period of approximately 30 s were displayed by the halimeter and recorded. The machine was allowed to return to 0 (about 60 to 120 s) before the next measurement was taken. Each cat was measured three times and a mean score calculated.

### Salivary pH

Salivary pH was measured using pH strips (Fisherbrand Plastic pH Strips; pH range = 0–14). All cats had their food withheld for at least 12 h prior to salivary pH measurement, using two strips on each side of the mouth per cat (4 total). Salivary pH for each cat was reported as the mean of 4 strips.

### Oral scores

Gingivitis, plaque, and calculus scoring was conducted by a blinded board-certified veterinarian according to a modified version of previous scoring systems (Mühlemann and Son, 1971; Gorrel et al., 1999). For each measurement, the canine, 3^rd^ premolar, and 4^th^ premolar teeth on the upper jaw (maxilla) and the canine, 3^rd^ premolar, 4^th^ premolar, and 1^st^ molar teeth on the lower jaw (mandible) were scored. To assess gingivitis, after an initial visual evaluation of the gingiva, a periodontal probe (Williams model, Cislak Manufacturing, Inc., Niles, IL) was placed subgingivally on the buccal side of each tooth, and values were assigned via visual assessment of inflammation and bleeding, if present, upon probing. Each tooth was graded by the average of the three scores obtained per tooth. Plaque levels were evaluated by using Trace Disclosing Solution (#231102, Young Dental, Algonquin, IL) to cover the teeth, followed by a gentle rinse of water to remove the excess. Plaque was revealed and subsequently scored for coverage and thickness according to Gorrel et al. (13) using the anatomical landmarks described in Hennet et al. (14) to divide the teeth into gingival and occlusal portions. The gingival and occlusal values for each tooth were averaged to obtain a tooth total score. Calculus scores were based on the visual assessment of coverage and thickness on the mesial, buccal, and distal portions of the tooth. The tooth score was the average of the scores for each of the three-tooth surfaces. Pocket depth was based on height from bottom of pocket to gingival margin: 1 = 0.0–0.5 mm, 2 = 0.5–1 mm, 3 = 1–1.5 mm, 4 = 1.5–2 mm, and 5 = 2–2.5 mm. The tooth score was the average pocket depth for each tooth.

### Plaque sample collection

Once teeth were scored, plaque samples were collected for microbiota and the teeth surfaces were cleaned. Teeth were assessed using a sterile periodontal probe on the gingival margin and sweeping along the base of the crown for supragingival samples, and by probing below the gingival margin for subgingival samples. Plaque samples were collected, and samples were placed into sterile 2.0 mL cryovials (Wheaton, Millville, NJ) and immediately placed on dry ice until storage at −80 °C, where they were stored until analysis.

### Plaque microbiota analysis

Plaque bacterial DNA was extracted according to the manufacturer’s instructions using the DNeasy PowerSoil Pro Kit (Qiagen, Valencia, CA) with bead beating using a vortex adaptor, followed by quantification of extracted DNA using a Qubit® 3.0 Fluorometer (Life Technologies, Grand Island, NY). DNA quality was determined using an E-Gel Power Snap Electrophoresis Device (Double Comb with SYBR, Invitrogen, Waltham, MA). 16S rRNA gene amplicons were generated using a Fluidigm Access Array (Fluidigm Corporation, South San Francisco, CA) in combination with Roche High Fidelity Fast Start Kit (Roche, Indianapolis, IN). The primers 515F (5’GTGCCAGCMGCCGCGGTAA-3′) and 806R (5’-GGACTACHVGGGTWTCTAAT-3′) that target a 252 bp-fragment of the V4 region of the 16S rRNA gene were used for amplification (primers synthesized by IDT Corp., Coralville, IA) (Caporaso et al., 2012). The CS1 forward and CS2 reverse tags were added according to the Fluidigm protocol. Quality of the amplicons was assessed using a Fragment Analyzer (Advanced Analytics, Ames, IA) to confirm amplicon region and size. A DNA pool was generated by combining equimolar amounts of the amplicons from each sample. The pooled samples were then size selected on a 2% agarose E-gel (Life technologies, Grand Island, NY) and extracted using a Qiagen gel purification kit (Qiagen, Valencia, CA). Cleaned size-selected pooled products were tested on an Agilent Bioanalyzer to confirm appropriate profile and average size. Illumina sequencing was performed on a MiSeq using v3 reagents (Illumina Inc., San Diego, CA) at the Roy J. Carver Biotechnology Center at the University of Illinois. Forward reads of Illumina 16S rRNA gene amplicon sequencing were trimmed using the FASTX-Toolkit (version 0.0.14), and the resulting sequence data were analyzed using QIIME2, version 2023.7 (15). Raw sequence amplicons were imported into the QIIME2 package and analyzed by the DADA2 pipeline for quality control (QC value ≥ 20) (16). Samples were then rarefied to 33,697 reads. Subsequent samples were assigned to taxonomic groups with the SILVA database (SILVA 138 99% ASV from 515F/806R region of sequences, with the QIIME2 classifier trained on 515F/806R V4 region of 16S) (17, 18). The taxonomic classifications produced by DADA2, as well as its quantifications, were imported into phyloseq (version 1.44.0) in R (version 4.3.1). The rarefied samples were used for alpha and beta diversity analysis. Principal coordinate analysis was performed using weighted and unweighted unique fraction metric (UniFrac) distances. Analysis of compositions of microbiomes with bias correction version 2 (ANCOM-BC2) was estimated using the ANCOMBC package (version 2.4.0) to identify specific taxa responsible for the observed discrimination between treatment samples. Benjamini-Hochberg adjusted *p* values were used, and *q* < 0.05 was considered statistically significant.

### Statistical analysis

Data were analyzed using the mixed model (PROC GLIMMIX) procedure of SAS (SAS Institute, Inc., Cary, NC). The fixed effects of diet were tested. Animals were considered a random effect. Least-squares means were calculated for each variable, with post-hoc pairwise comparisons adjusted using the Tukey method. Statistical significance was determined at a threshold of *p* < 0.05, and pooled standard errors of the mean (SEM) were reported.

## Results

Food intake averaged 51.95 ± 2.6 g/day for cats fed dry diet and 156.7 ± 6.7 g/day for those fed wet diet. Body weight (*p* = 0.7568) and body condition score (*p* = 0.8950) remained stable throughout the study and did not differ between dietary treatments (data not shown).

**Figure 1:**
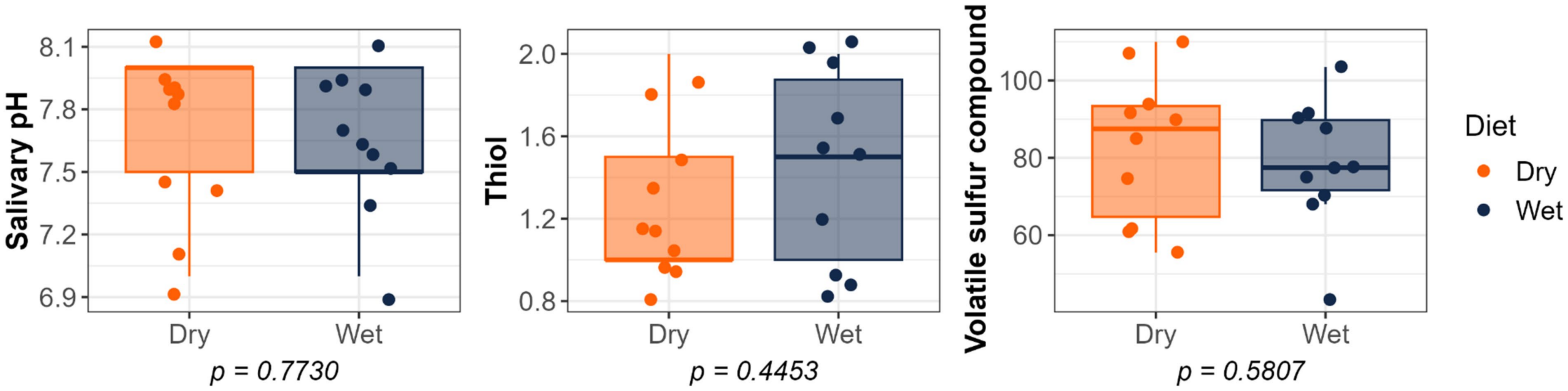
Salivary pH, thiol concentrations, and volatile sulfur compound concentrations of cats fed commercial wet or dry diets.

**Figure 2:**
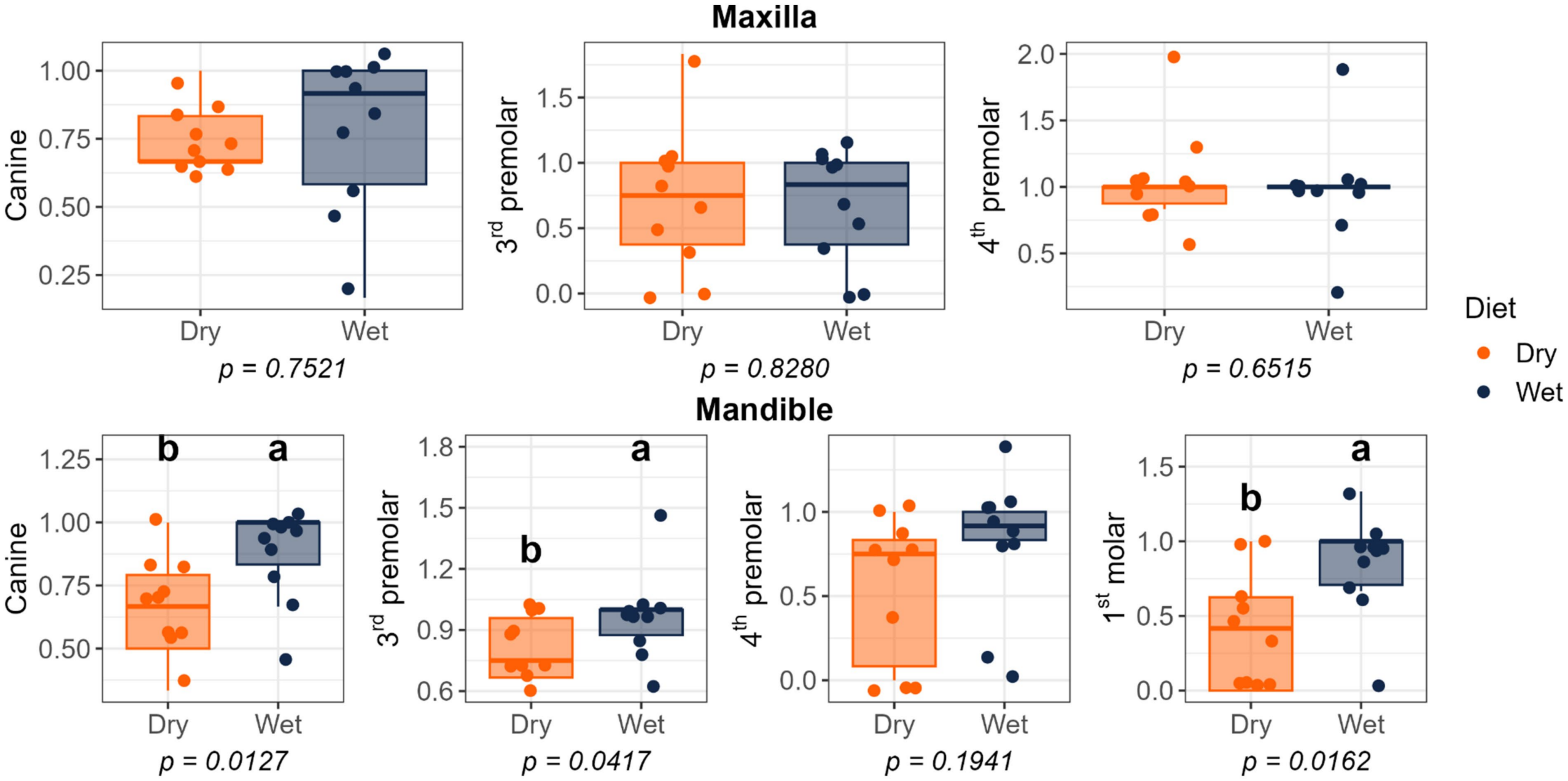
Gingivitis scores of cats fed commercial wet or dry diets. Scores reflect the severity of gingival inflammation based on clinical presentation: 0 = no gingivitis; 1 = very mild (‘incipient’) gingivitis characterized by redness and swelling without bleeding on probing; 2 = mild gingivitis with redness, swelling, and delayed bleeding on probing; 3 = moderate gingivitis with redness, swelling, and immediate bleeding on probing; 4 = severe gingivitis with ulceration, spontaneous hemorrhage, and profuse bleeding on probing.

**Figure 3:**
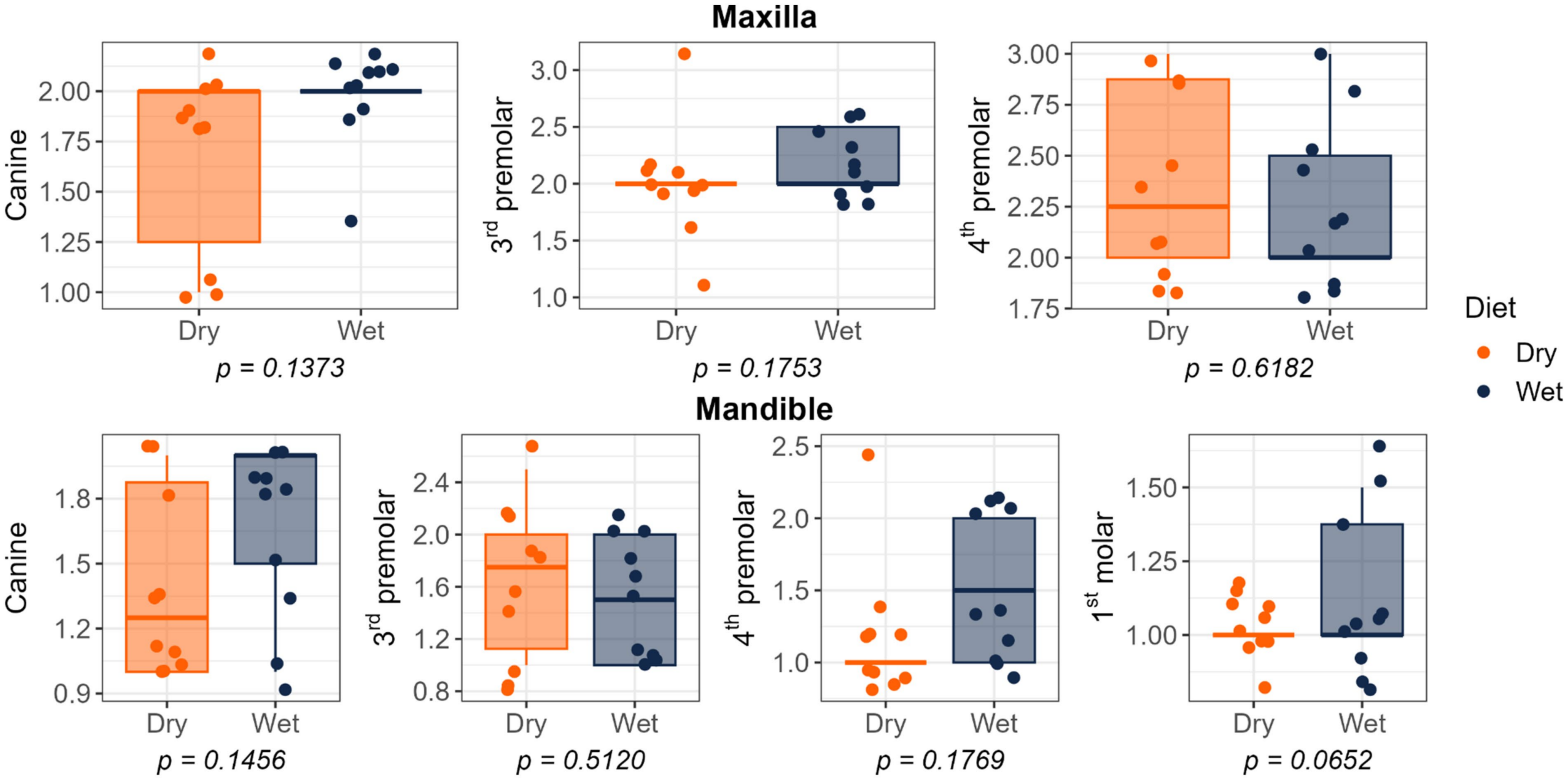
Periodontal pocket depth scores of cats fed commercial wet or dry diets. Scores indicate the depth of the gingival sulcus or periodontal pocket as follows: 1 = 0.0–0.5 mm; 2 = 0.5–1.0 mm; 3 = 1.0–1.5 mm; 4 = 1.5–2.0 mm; 5 = 2.0–2.5 mm.

**Figure 4:**
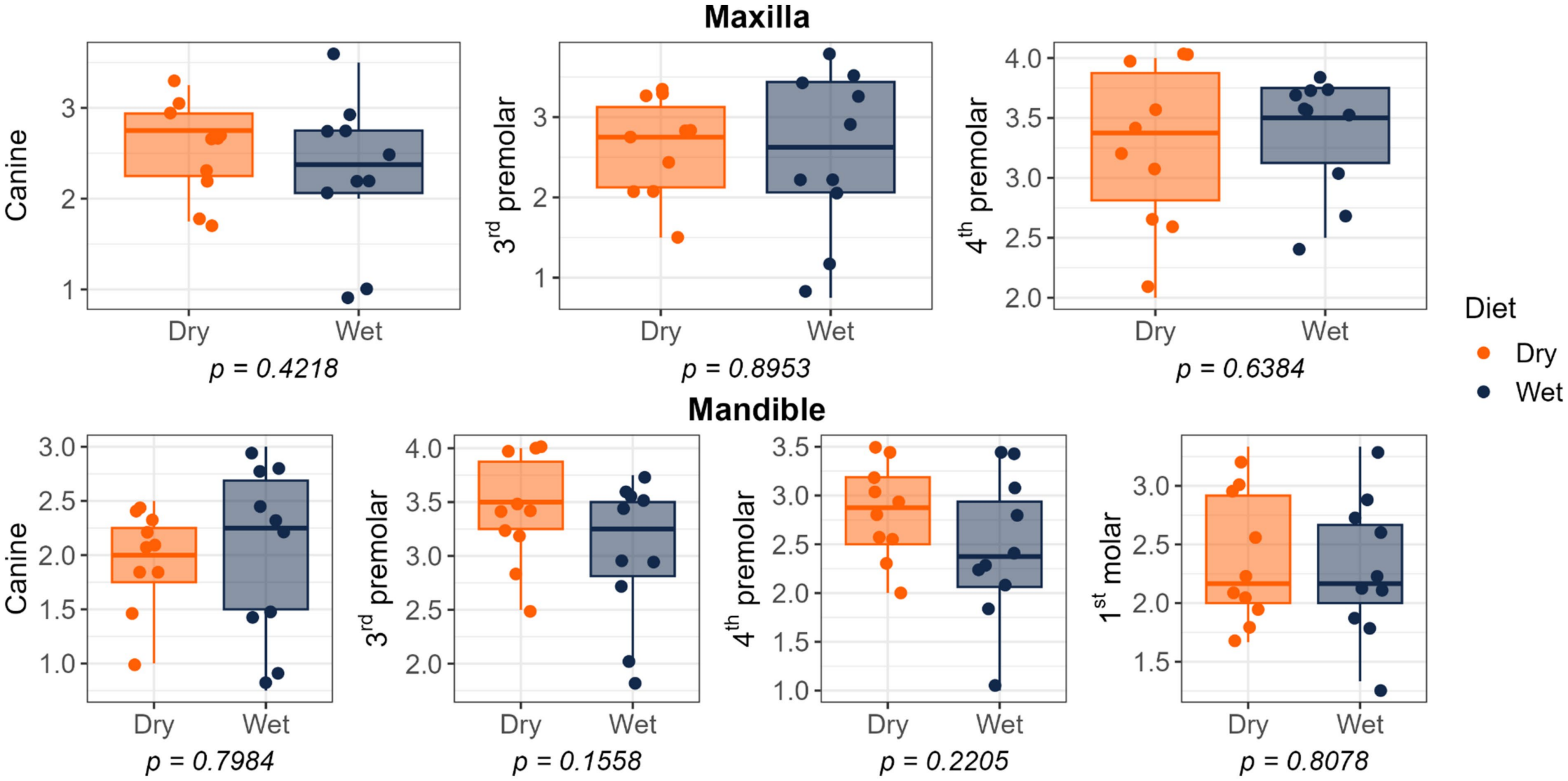
Plaque coverage scores of cats fed commercial wet or dry diets. Scores represent the extent of plaque coverage on the buccal tooth surface, defined as follows: 0, no detectable plaque; 1, scattered plaque covering less than 24%; 2, plaque covering 25 to 49%; 3, plaque covering 50 to 74%; and 4, plaque covering more than 75%.

**Figure 5:**
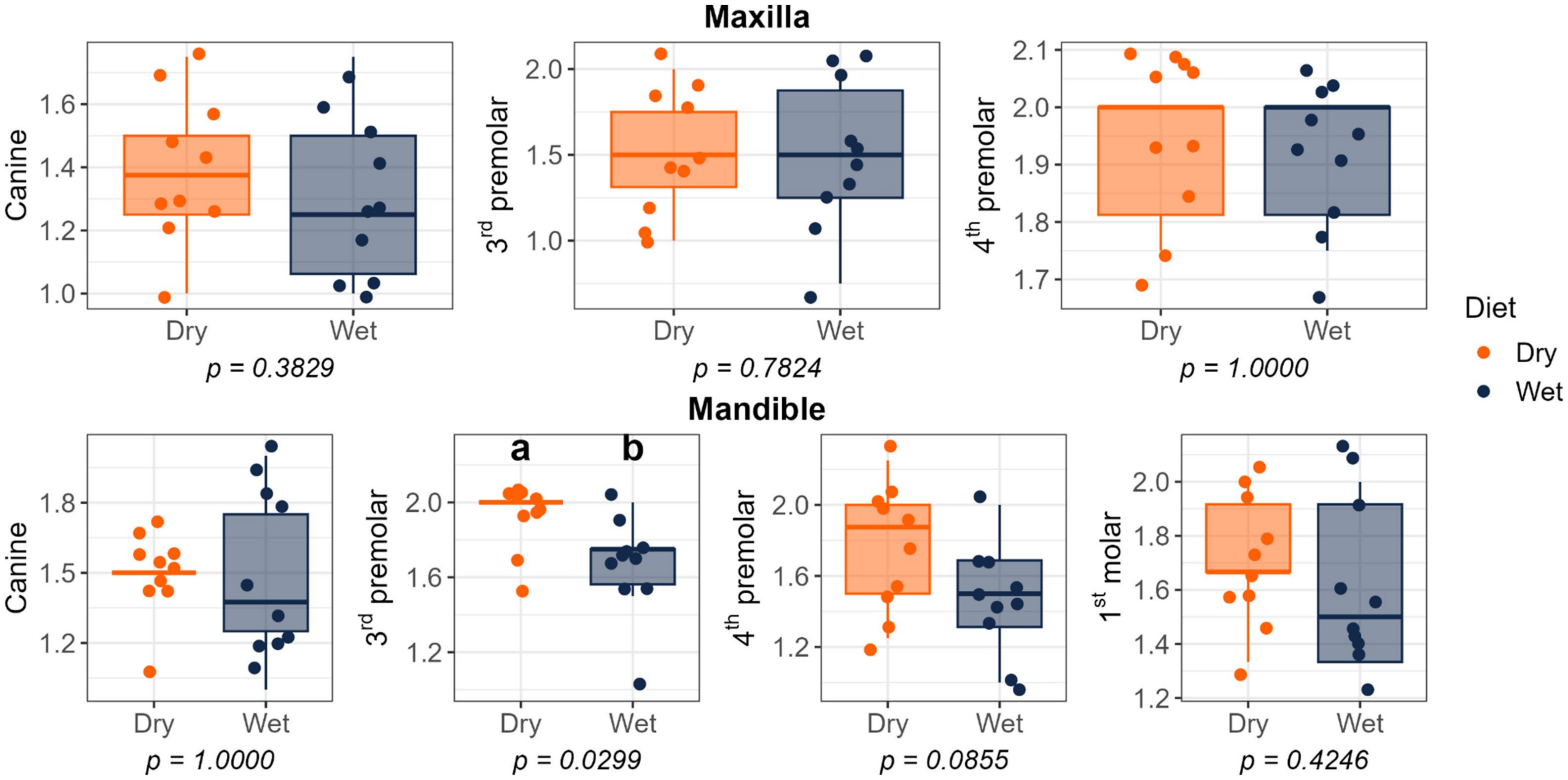
Plaque thickness scores of cats fed commercial wet or dry diets. Scores represent the severity of plaque deposits as follows: 1 = Light; 2 = Moderate; 3 = Heavy.

**Figure 6:**
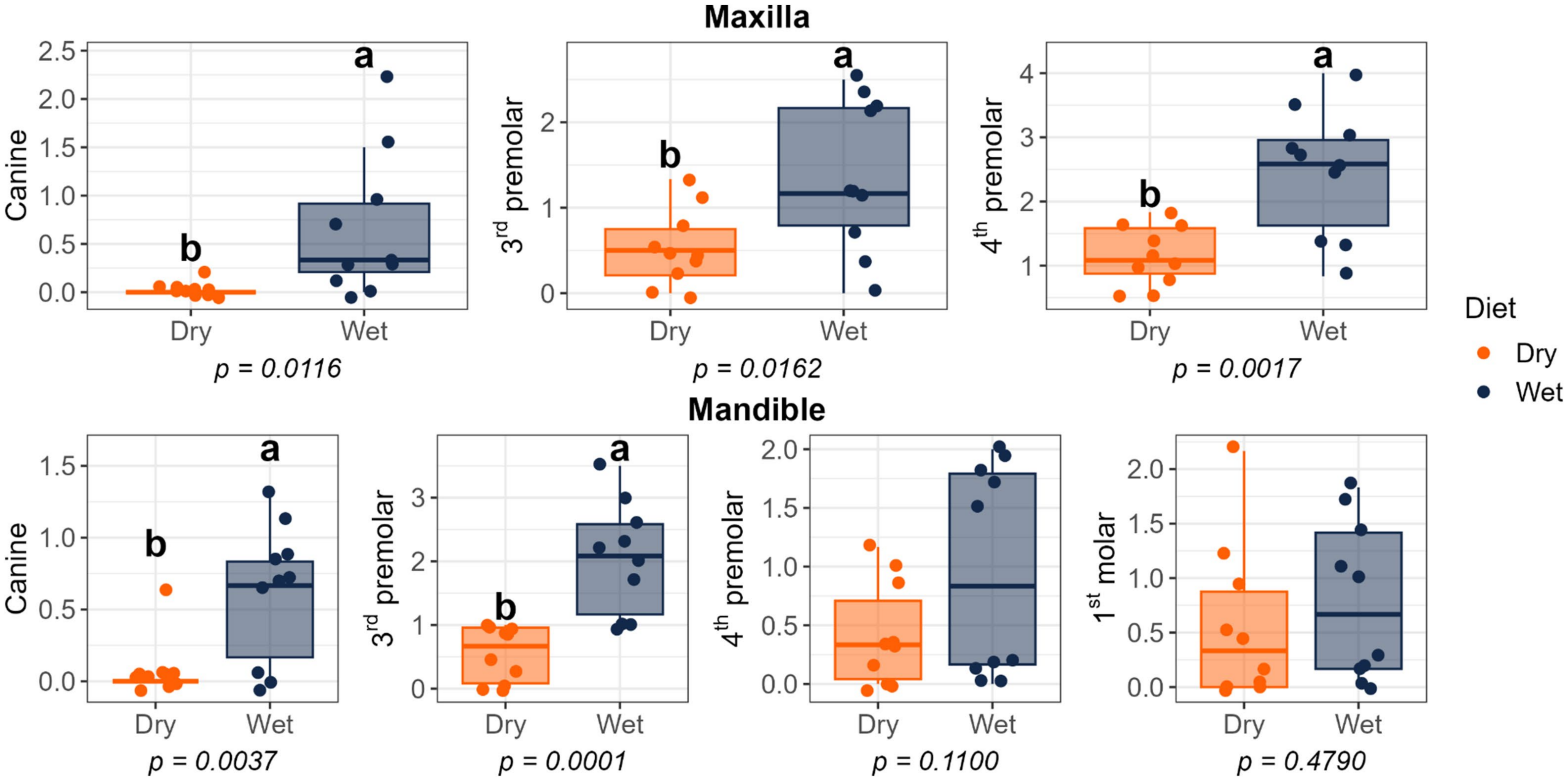
Calculus coverage scores of cats fed commercial wet or dry diets. Scores indicate the percentage of the buccal tooth surface covered by calculus: 0 = no detectable calculus; 1 = scattered calculus covering less than 24%; 2 = calculus covering 25–49%; 3 = calculus covering 50–74%; 4 = calculus covering more than 75%.

**Figure 7:**
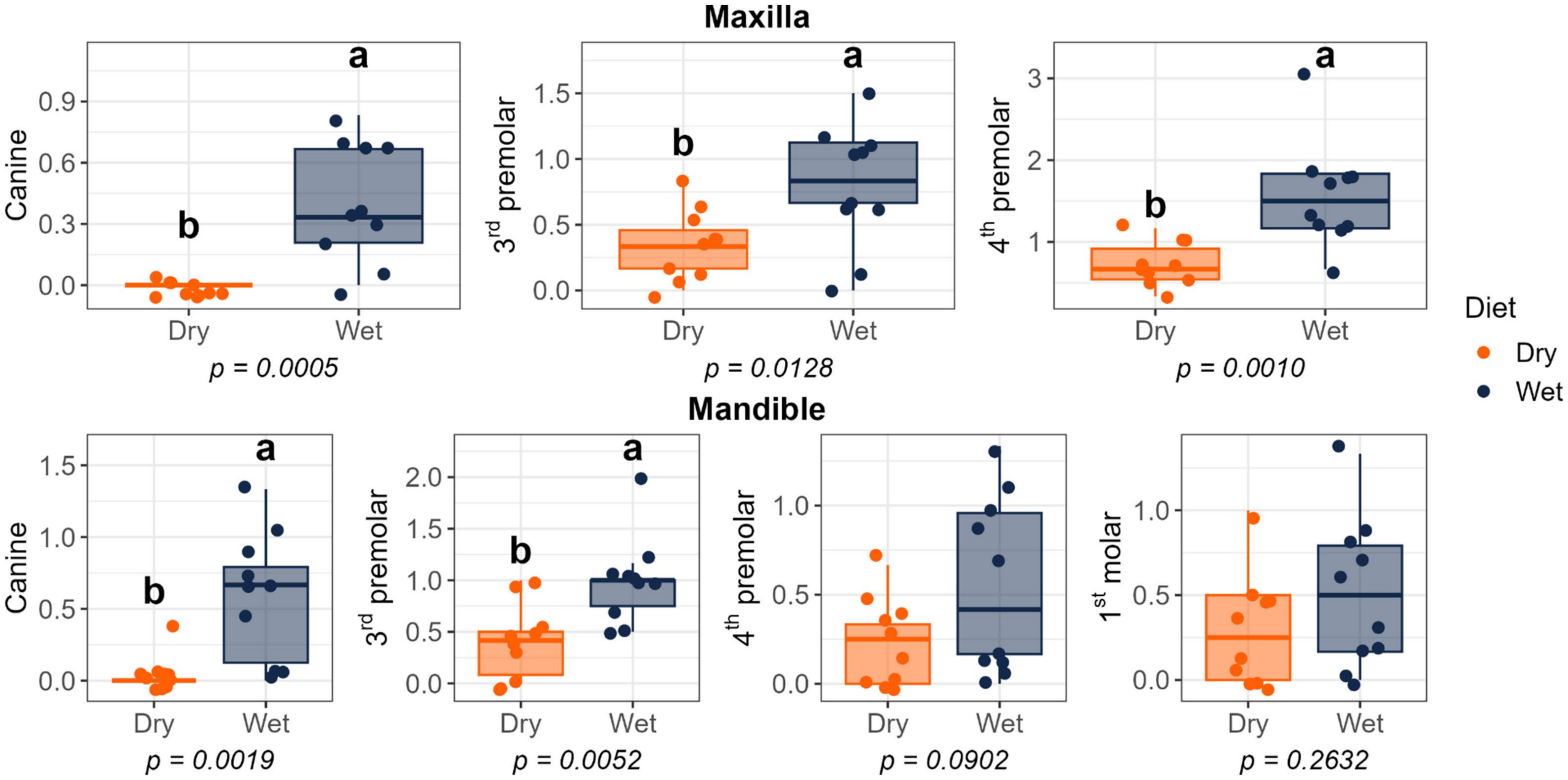
Calculus thickness scores of cats fed commercial wet or dry diets. Scores represent the thickness of calculus deposits as follows: 1 = thickness less than 0.5 mm; 2 = thickness between 0.5 and 1.0 mm; 3 = thickness greater than 1.0 mm.

Dietary treatment had no effect on salivary pH, thiol concentrations, or volatile sulfur compound concentrations (Figure 1). However, cats fed the wet diet had higher (*p* < 0.05) gingivitis scores on the mandibular canine, 3rd premolar, and 1st molar teeth than those fed the dry diet (Figure 2). Pocket scores (Figure 3) and plaque coverage (Figure 4) were not impacted by dietary treatment. Plaque thickness was greater (*p* < 0.05) on the mandibular 3rd premolar teeth of cats fed the wet diet than those fed the dry diet (Figure 5). Calculus coverage and thickness on the maxillary canine, 3rd premolar, and 4th premolar teeth and mandibular canine and 3rd premolar teeth were higher (*p* < 0.05) in cats fed the wet diet than those fed the dry diet (Figures 6, 7).

**Figure 8:**
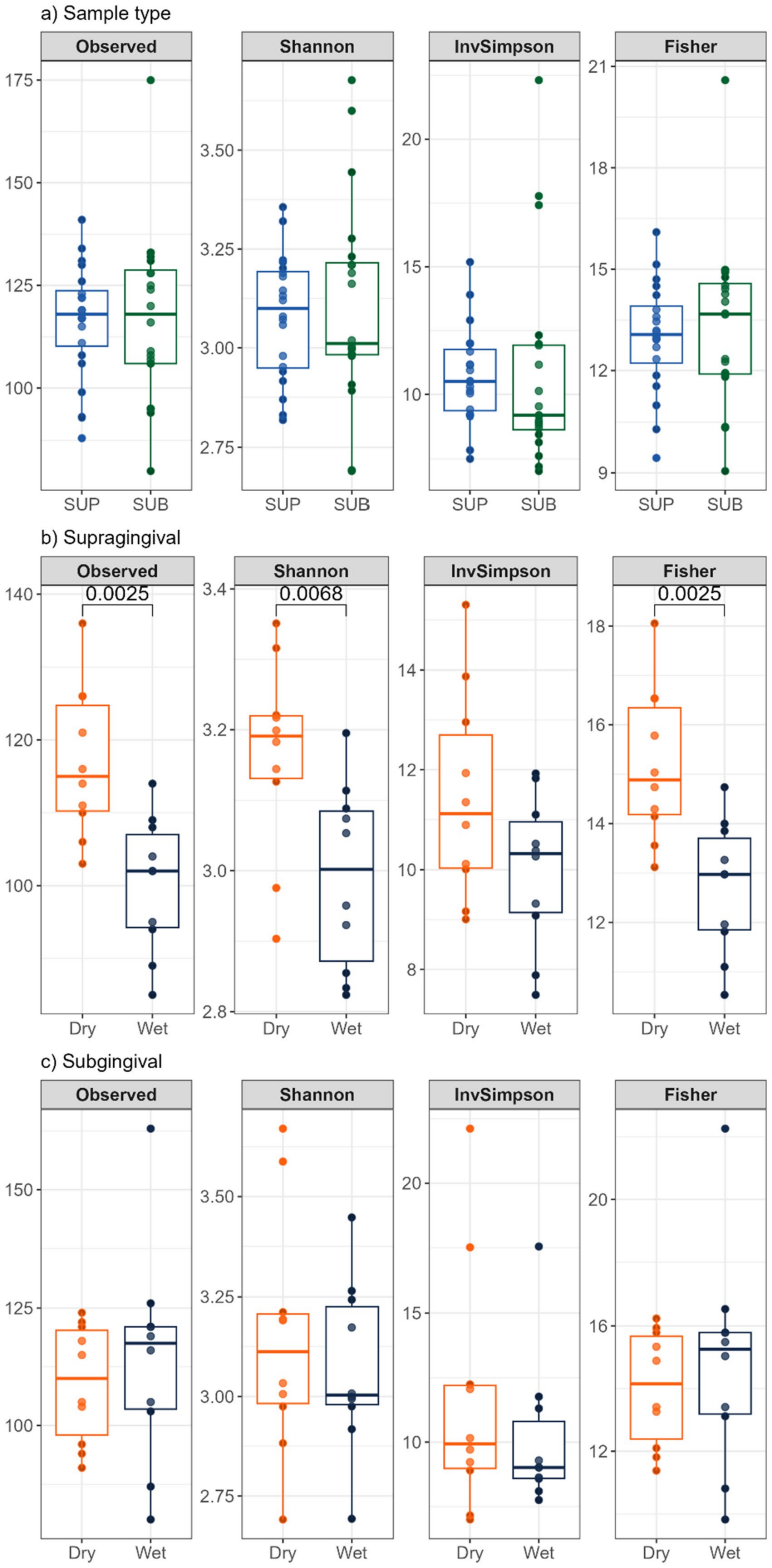
Bacterial alpha diversity measures of plaque samples from cats fed commercial wet or dry diets: **(a)** supragingival (SUP) vs. subgingival (SUB) plaque, **(b)** wet vs. dry diets within supragingival samples, and **(c)** wet vs. dry diets within subgingival samples. Observed = observed features, Shannon = Shannon Index, InvSimpson = inverse Simpson Index, Fisher = Fisher Index.

**Figure 9:**
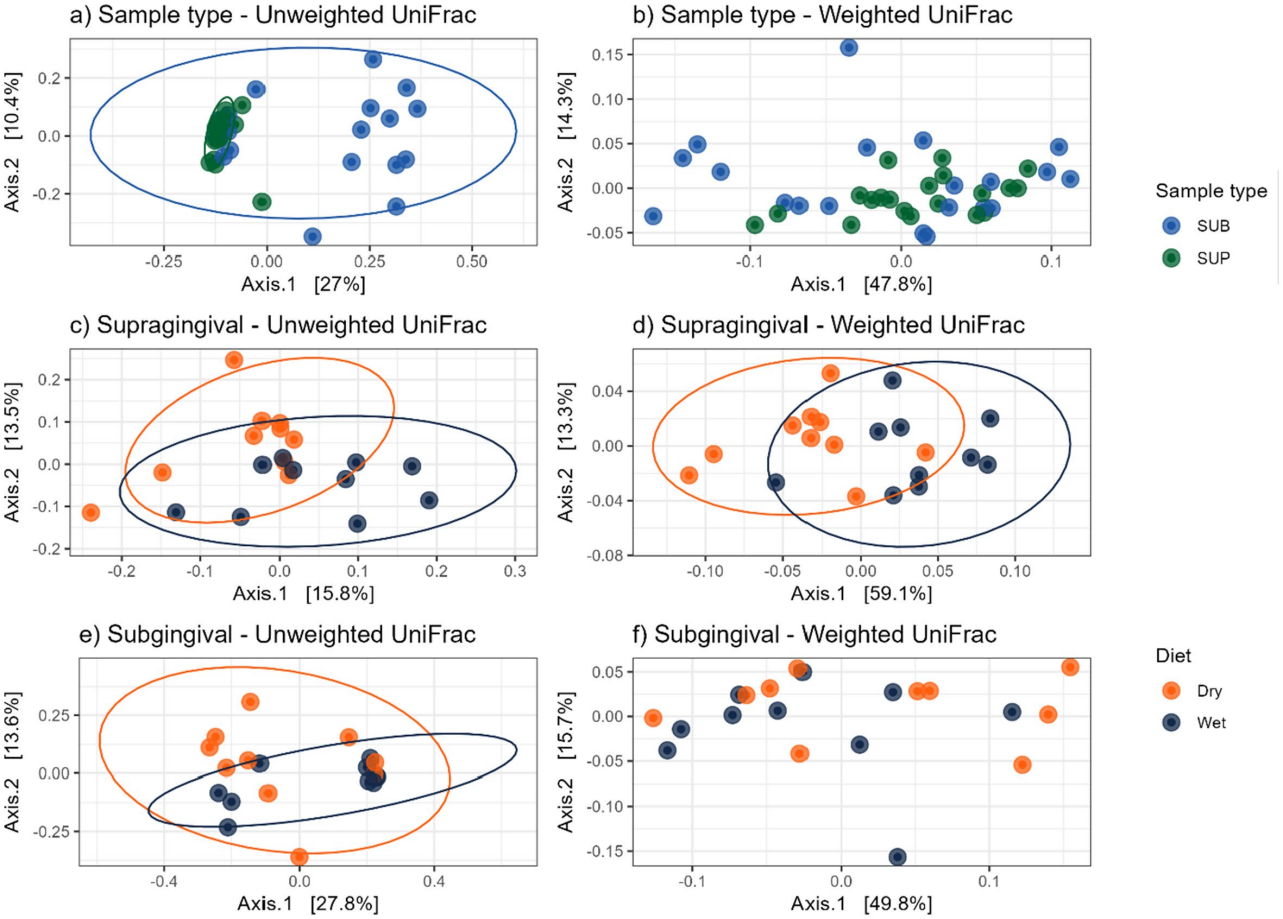
Bacterial beta diversity measures of plaque samples from cats fed wet or dry diets, shown by principal coordinate analysis plots based on UniFrac distances: **(a)** unweighted and **(b)** weighted UniFrac distances comparing supragingival (SUP) and subgingival (SUB) samples; **(c)** unweighted and **(d)** weighted UniFrac distances comparing supragingival plaque samples of cats fed dry or wet diets; **(e)** unweighted and **(f)** weighted UniFrac distances comparing subgingival plaque samples of cats fed dry or wet diets.

Bacterial alpha diversity measures (i.e., observed features, Shannon Index, Fisher Index) were lower (*p* < 0.05) in supragingival samples of cats fed the wet diet than those fed the dry diet, but no differences were detected between sample sites or within diet of subgingival samples (Figure 8). Bacterial beta diversity analysis according to unweighted UniFrac distances demonstrated separation between supragingival and subgingival samples. Beta diversity analysis using unweighted UniFrac revealed differences between cats fed wet and dry diets in both sample sites, while weighted UniFrac distances only detected diet-related differences in supragingival samples (Figure 9).

**Figure 10:**
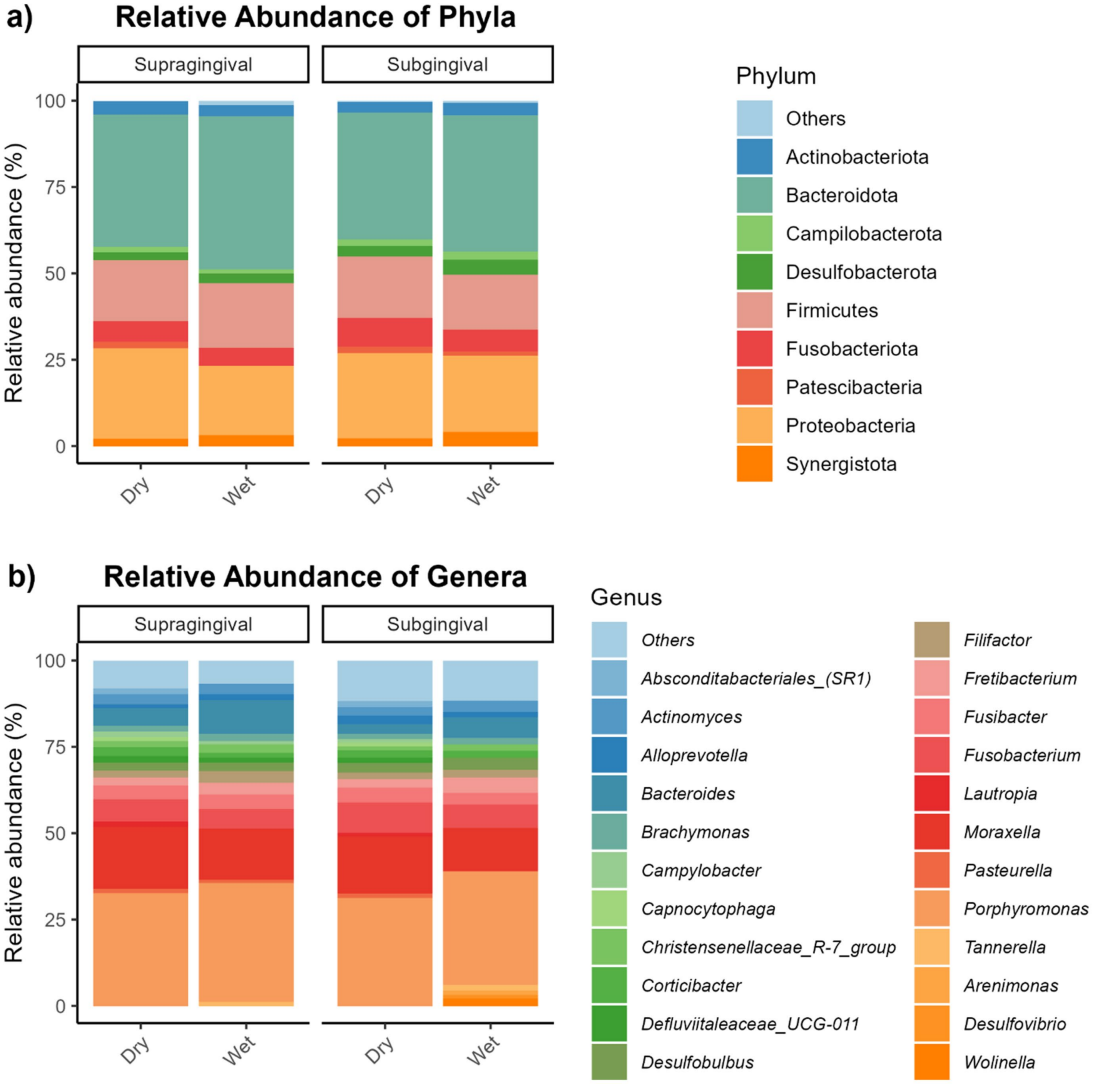
Relative abundances of bacterial taxa at the phylum and genus levels in supragingival and subgingival plaque samples from cats fed dry or wet diets. Panels show phylum-level composition **(a)** and genus-level composition **(b)** for supragingival and subgingival samples. Taxa with relative abundance ≤1% across all samples were grouped under “Others.”

Bacteroidota and Proteobacteria were the predominant bacterial phyla, while *Porphyromonas* and *Moraxella* were the most abundant bacterial genera, in supragingival and subgingival samples (Figure 10). Relative abundances of Bacteroidota, *Bacteroides*, *Campylobacter*, and *Christensenellaceae R-7 group* were more (*p* < 0.05) abundant in supragingival plaque samples than subgingival plaque samples, whereas Fusobacteriota, *Wolinella*, *Fusobacterium*, *Luteimonas*, and *Conchiformibius* relative abundances were greater (*p* < 0.05) in subgingival plaque samples than supragingival samples (Table 2). Compared with cats fed the dry diet, those fed the wet diet had higher (*p* < 0.05) relative abundances of Bacteroidota and *Bacteroides* in supragingival plaque samples. In contrast, cats fed the dry diet had higher (*p* < 0.05) relative abundances of Proteobacteria, *Streptococcus*, *Luteimonas*, and *Lautropia* than those fed the wet diet (Table 3). In subgingival plaque samples, the relative abundances of Synergistota, Bacteroides, *Fretibacterium*, *Campylobacter*, and the *Christensenellaceae R-7 group* were greater (*p* < 0.05) in cats fed the wet diet than those fed the dry diet (Table 4).

**Table 2:** Relative abundances (% of sequences) of predominant bacterial phyla and genera in the supragingival and subgingival plaque of cats.

Phyla	Genera	SUP^1^	SUB^2^	SEM^3^	*p*-value
Bacteroidota		41.27^a^	36.78^b^	1.73	0.0325
	*Bacteroides*	6.78^a^	3.99^b^	0.82	0.0023
	*Porphyromonas*	30.96	27.79	1.73	0.1689
	*Alloprevotella*	1.34	1.60	0.47	0.5826
Campilobacterota		1.46	2.11	0.35	0.1824
	*Wolinella*	0.24^b^	1.32^a^	0.34	0.0295
	*Campylobacter*	1.23^a^	0.78^b^	0.12	0.0032
Chloroflexi		0.30	0.38	0.10	0.5595
	*Flexilinea*	0.30	0.38	0.10	0.5595
Firmicutes		18.14	16.88	0.72	0.2149
	*Christensenellaceae_R-7_group*	1.87^a^	1.28^b^	0.19	0.0098
	*Fastidiosipila*	0.06	0.19	0.08	0.1376
	*Fusibacter*	3.76	3.33	0.24	0.1581
	*Proteocatella*	0.49	0.56	0.12	0.6342
	*Streptococcus*	0.21	0.18	0.12	0.8313
Fusobacteriota		5.62^b^	7.30^a^	0.51	0.0128
	*Fusobacterium*	5.59^b^	7.02^a^	0.55	0.0404
Proteobacteria		23.10	24.62	2.45	0.6028
	*Moraxella*	15.06	13.29	1.78	0.4351
	*Lautropia*	0.91	1.10	0.39	0.7078
	*Neisseria*	0.42	0.46	0.13	0.8094
	*Pasteurella*	1.08	0.89	0.24	0.3576
	*Luteimonas*	0.19^b^	0.58^a^	0.11	0.0054
	*Arenimonas*	0.29	1.12	0.32	0.0767
	*Corticibacter*	1.90	1.97	0.37	0.8874
	*Conchiformibius*	0.06^b^	0.44^a^	0.13	0.0364
Synergistota		2.66	3.18	0.46	0.1291
	*Fretibacterium*	2.64	3.16	0.46	0.1286

^1^SUP, supragingival plaque.

^2^SUB, subgingival plaque.

^3^SEM, pooled standard error of the means.

^a,b^Mean values within the same row with unlike superscript letters differ (*P* < 0.05).

**Table 3:** Relative abundances (% of sequences) bacterial phyla and genera in the supragingival plaque of cats fed commercial wet or dry diets.

Phyla	Genera	Dry	Wet	SEM^1^	*p*-value
Bacteroidota		38.16^b^	44.39^a^	1.44	0.0067
	*Bacteroides*	4.54^b^	9.01^a^	1.12	0.0112
	*Porphyromonas*	29.83	32.08	1.26	0.2223
	*Alloprevotella*	1.06	1.62	0.54	0.4691
Campilobacterota		1.65	1.28	0.19	0.1802
	*Wolinella*	0.20	0.28	0.08	0.5349
	*Campylobacter*	1.45	1.00	0.16	0.0626
Chloroflexi		0.20	0.40	0.08	0.1079
	*Flexilinea*	0.20	0.40	0.08	0.1079
Firmicutes		17.65	18.63	0.66	0.3122
	*Christensenellaceae_R-7_group*	1.56	2.17	0.25	0.1114
	*Fastidiosipila*	0.07	0.05	0.04	0.7124
	*Fusibacter*	3.66	3.87	0.21	0.4792
	*Proteocatella*	0.60	0.38	0.11	0.1574
	*Streptococcus*	0.40^a^	0.01^b^	0.13	0.0428
	*Dielma*	0.05	0.00	0.03	0.3025
Fusobacteriota		5.97	5.27	0.55	0.3810
	*Fusobacterium*	5.94	5.24	0.55	0.3795
Proteobacteria		26.17^a^	20.03^b^	1.59	0.0139
	*Moraxella*	16.35	13.77	1.27	0.1692
	*Lautropia*	1.44^a^	0.37^b^	0.26	0.0088
	*Neisseria*	0.53	0.32	0.13	0.2778
	*Pasteurella*	1.21	0.96	0.36	0.6247
	*Luteimonas*	0.31^a^	0.07^b^	0.05	0.0023
	*Arenimonas*	0.41	0.17	0.12	0.1810
	*Corticibacter*	2.45	1.35	0.40	0.0695
	*Conchiformibius*	0.08	0.04	0.03	0.3906
Synergistota		2.13	3.19	0.56	0.1996
	*Fretibacterium*	2.12	3.17	0.56	0.2050

^1^SEM, pooled standard error of the means.

^a,b^Mean values within the same row with unlike superscript letters differ (*P* < 0.05).

**Table 4:** Relative abundances (% of sequences) of bacterial phyla and genera in the subgingival plaque of cats fed commercial wet or dry diets.

Phyla	Genera	Dry	Wet	SEM^1^	*p*-value
Bacteroidota		34.70	38.86	2.99	0.3391
	*Bacteroides*	2.56^b^	5.43^a^	0.91	0.0382
	*Porphyromonas*	26.39	29.18	52.22	0.9710
	*Prevotellaceae_Ga6A1_group*	0.16	0.02	0.11	0.3875
	*Alloprevotella*	1.84	1.36	0.78	0.6697
Campilobacterota		1.90	2.31	0.68	0.6800
	*Wolinella*	0.87	1.78	0.67	0.3494
	*Campylobacter*	1.04^b^	0.53^a^	0.15	0.0267
Chloroflexi		0.20	0.57	0.17	0.1467
	*Flexilinea*	0.20	0.57	0.17	0.1467
Firmicutes		18.11	15.64	1.26	0.1803
	*Christensenellaceae_R-7_group*	0.88^b^	1.67^a^	0.24	0.0294
	*Faecalibacterium*	0.26	0.00	0.16	0.2557
	*Fastidiosipila*	0.29	0.08	0.15	0.3440
	*Fusibacter*	3.71	2.96	0.44	0.2421
	*Proteocatella*	0.63	0.49	0.21	0.6518
	*Streptococcus*	0.36	0.01	0.19	0.2131
Fusobacteriota		8.21	6.39	0.84	0.1421
	*Fusobacterium*	7.94	6.10	0.92	0.1740
Proteobacteria		26.12	23.11	4.63	0.6516
	*Moraxella*	14.80	11.77	3.35	0.5310
	*Lautropia*	1.24	0.96	0.74	0.7921
	*Neisseria*	0.46	0.46	0.22	0.9968
	*Pseudomonas*	0.06	0.03	0.04	0.5945
	*Pasteurella*	0.97	0.80	0.35	0.7389
	*Luteimonas*	0.78	0.37	0.20	0.1619
	*Arenimonas*	1.06	1.18	0.65	0.8958
	*Corticibacter*	2.07	1.87	0.63	0.8250
	*Conchiformibius*	0.36	0.52	0.26	0.6643
Synergistota		2.16^b^	4.21^a^	0.66	0.0420
	*Fretibacterium*	2.15^b^	4.17^a^	0.66	0.0440

^1^SEM, pooled standard error of the means.

^a,b^Mean values within the same row with unlike superscript letters differ (*P* < 0.05).

**Figure 11:**
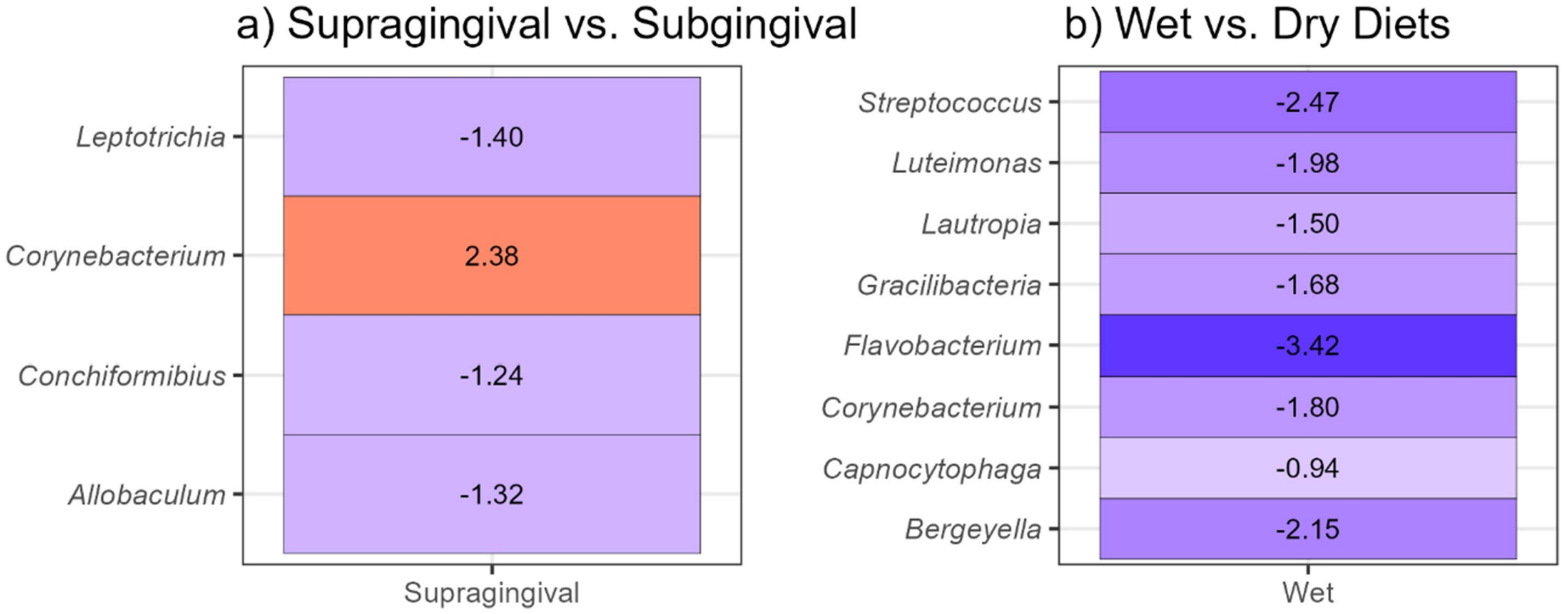
Analysis of composition of microbiomes with bias correction (ANCOM-BC2) identified differentially abundant bacterial genera of feline plaque samples (*q* < 0.05): **(a)** supragingival vs. subgingival plaque samples, **(b)** supragingival samples of cats fed a wet vs. a dry diet.

Analysis of composition of microbiomes with bias correction (ANCOM-BC2) revealed that the relative abundances of *Leptotrichia*, *Conchiformibius*, and *Allobaculum* were lower (*p* < 0.05) and the relative abundance of *Corynebacterium* was higher (*p* < 0.05) in supragingival plaque than in subgingival plaque. Additionally, the relative abundances of *Bergeyella*, *Capnocytophaga*, *Corynebacterium*, *Flavobacterium*, *Gracilibacteria*, *Lautropia*, *Luteimonas*, and *Streptococcus* were lower (*p* < 0.05) in supragingival plaque samples of cats fed the wet diet than those fed the dry diet (Figure 11). No diet-related differences were observed in subgingival plaque samples.

## Discussion

Insights into the feline oral microbiome and the factors that shape its composition are still relatively limited. In our study, the overall bacterial profile, particularly the distribution of dominant phyla and the detection of key genera, closely aligned with previously reported patterns. The dominant phyla included Bacteroidetes, Proteobacteria, and Firmicutes, with *Porphyromonas* identified as the most prevalent genus (10, 19, 20).

To our knowledge, this study is the first to compare supragingival and subgingival plaque samples in cats. Supragingival plaque, which accumulates on the tooth surface above the gum line, is characterized by a high abundance of gram-positive aerobic bacteria. Its development is a prerequisite for the establishment of subgingival plaque within the gingival sulcus, where gram-negative anaerobic bacteria are highly abundant. Periodontal disease results from both the direct effects of these microorganisms and their byproducts on periodontal tissues, and the indirect effects mediated by the host immune response, leading to inflammation and tissue destruction (21, 22). Thus, distinguishing the differences between these sites may provide valuable insights into feline oral health.

In dogs, bacterial alpha diversity measures (i.e., observed features, Shannon Index, Faith’s phylogenetic diversity) have been reported to be higher in subgingival than supragingival samples. However, no distinct separation between subgingival and supragingival sites was observed in beta diversity analyses using unweighted and weighted UniFrac distances. Taxonomic differences were evident, with higher relative abundances of Actinobacteria, Proteobacteria, *Corynebacterium*, *Enhydrobacter,* and *Moraxella* in supragingival samples. In contrast, subgingival samples were enriched in Bacteroidetes, Firmicutes, Spirochaetes *Porphyromonas*, *Fusibacter*, *Fusobacterium*, *Treponema,* and *Campylobacter* (23). Another study in dogs demonstrated that the subgingival habitat harbors significantly higher proportions of *Prevotella intermedia*, *Streptococcus constellatus*, *Campylobacter rectus*, and *Campylobacter showae*, whereas the proportion of *Actinomyces naeslundii* was significantly lower. Additionally, lower proportions of *Parvimonas micra*, *Fusobacterium nucleatum* ss. *polymorphum*, and *Streptococcus intermedius* were observed in supragingival plaque (24).

In contrast to what has been reported in dogs, the present study did not detect any differences in alpha diversity measures between the supragingival and subgingival samples of cats. However, beta diversity analysis revealed clear clustering by sample type, indicating distinct microbial community compositions between the two sites. Notably, *Campylobacter* was enriched in supragingival samples, which contrasts with the findings in dogs. In agreement with the canine data, *Fusobacterium* was enriched in subgingival samples. Moreover, several bacterial taxa differed between supragingival and subgingival samples in cats that were not reported in dog studies, underscoring the need for further studies in cats that may identify the core oral bacterial communities.

Regarding dietary effects, a previous study reported that cats fed a dry diet had higher oral bacterial alpha diversity measures than those fed a wet diet. Cats fed a dry diet also had an enrichment of bacterial genera such as *Actinobacillus*, *Acholeplasma*, *Treponema*, and *Porphyromonas*, while cats fed a wet diet had higher levels of *Proteobacteria* in their dental plaque (10). In dogs, dry diet consumption was similarly associated with increased abundances of bacterial taxa linked with oral health, including *Pasteurella*, *Capnocytophaga*, and *Corynebacterium*, lower abundances of known oral pathogens such as *Fretibacterium fastidiosum*, *Filifactor alocis*, *Treponema medium*, *Tannerella forsythia*, *Porphyromonas canoris*, and *Porphyromonas gingivalis*, and higher bacterial alpha diversity measures in supragingival plaque (2).

Similarly, in the present study, cats fed a dry diet had greater alpha diversity in their supragingival plaque microbiomes. Furthermore, *Capnocytophaga* and *Corynebacterium* had similar enrichment patterns as those reported previously. Additionally, we observed increased abundances of genera such as *Bergeyella*, *Flavobacterium*, *Gracilibacteria*, *Lautropia*, *Luteimonas*, and *Streptococcus* in the supragingival plaque of cats fed a dry diet, whereas *Bacteroides* was more abundant in the supragingival plaque of cats fed the wet diet. Notably, *Capnocytophaga* and *Bergeyella* have been described as dominant genera in the healthy feline oral cavity (19, 25), and also were identified as a core oral microbiome in cats (19, 20). In healthy cats, these taxa, along with *Corynebacterium*, *Flavobacterium*, *Lautropia*, *Luteimonas*, and *Streptococcus*, are present at higher abundances than cats affected by chronic gingivostomatitis, which have increased *Porphyromonas*, *Bacteroides*, *Treponema*, *Tannerella*, *Peptostreptococcus*, and *Fusobacterium* abundances (26). Collectively, our data indicate that consumption of a dry diet is associated with a higher oral bacterial alpha diversity and a higher relative abundance of health-associated bacterial taxa in feline supragingival plaque, suggesting potential benefits for oral health.

Calculus formation and gingivitis development are critical factors in the progression of periodontal disease, as both increase as oral health declines. The close relationship between the extent of calculus and gingivitis reflects worsening dental health. Dental calculus is a hardened, mineralized form of plaque that develops when plaque is exposed to calcium and phosphate ions in saliva and crevicular fluid. Mineralization can begin within 48 h of plaque accumulation. Calculus may form both supragingivally and subgingivally, and its development is influenced by oral pH and diet. While bacterial plaque is the primary cause of periodontal disease, calculus contributes by providing a rough surface that promotes bacterial adhesion, plaque accumulation, and irritation of the gingival tissues (3, 27). Studies have shown that dental calculus and plaque are less common in cats fed dry diets than those fed wet diets (8). Similarly, dogs fed dry extruded diets have reduced plaque buildup and improved breath odor, supporting the potential oral health benefits of dry diet (2). This difference is often attributed to the abrasive nature of dry diet, which aids in mechanically removing dental plaque, whereas wet or soft diets have been linked to poorer dental health outcomes (8, 28). Additionally, consumption of a dry diet stimulates greater saliva production, which contains immunoglobulins that can enhance the oral immune response and help prevent dental problems (29). Furthermore, incisors in young and adult cats fed dry diets were less likely to have high oral health scores than premolars and molars (cheek teeth) in older cats consuming diets with a wet component. Cheek teeth generally scored higher, even in younger cats, but predominantly when fed wet commercial or homemade (soft) diets. Overall, diet and tooth type were identified as key factors driving variability in oral health scores and thus significantly influencing feline oral health status (9).

In the present study, no differences in salivary pH were observed between dietary treatments; however, consistent with these findings, cats fed a wet diet had higher calculus scores at multiple sites on the maxilla and mandible (including canines and premolars), along with increased gingivitis scores on the mandible at the canines, premolars, and molars. Interestingly, plaque accumulation was greater on the mandibular premolars of cats consuming dry diets. This difference may be explained by the anatomical characteristics of premolars and molars, which are larger teeth positioned deeper within the cheek, making it more difficult for the tongue to clean them effectively. This environment promotes food retention, encouraging bacterial growth and increasing the risk of plaque and gingivitis. Moreover, premolars and molars, which play a primary role in mastication, often have diastemata (i.e., space between teeth) more frequently than incisors, further facilitating food entrapment. In contrast, incisors not only assist in biting but also contribute to grooming and self-cleaning behaviors, resulting in greater abrasion that helps reduce plaque formation (9).

This study had some limitations, including a small sample size and a focus on a single cat breed. Future research should explore a wider range of dietary formats, including differences in moisture content and processing methods, to better understand how variations in texture and density influence oral health outcomes and microbiota composition. Additionally, investigating the effects of varying protein, carbohydrate, and fat concentrations may provide insight into how macronutrient content shapes oral microbial populations. Lastly, because cat breeds differ in jaw structure, tooth spacing, orientation, and potentially their susceptibility to periodontal disease, it is recommended that future studies include multiple breeds to capture this variability.

Although many oral health measures did not differ between diet groups, this study showed that cats fed a dry diet had reduced calculus accumulation and a more diverse oral microbiome enriched with bacteria associated with oral health and a lower risk of periodontal disease than those fed a wet diet. These results indicate that diets commonly fed to domestic cats in the United States can significantly influence the composition and diversity of the feline oral microbiota. Nevertheless, further research is needed to understand how these microbial changes affect biofilm development, plaque and calculus buildup, and the progression of periodontal disease in cats.

## Data Availability

The original contributions presented in the study are publicly available. These data can be found here: https://www.ncbi.nlm.nih.gov/genbank/, SRA: SRP627208 and BioProject: PRJNA1334357.
